# Antenatal Betamethasone Induces Increased Surfactant Proteins and Decreased Foxm1 Expressions in Fetal Rabbit Pups

**DOI:** 10.7150/ijms.62286

**Published:** 2021-07-25

**Authors:** Yong-Sung Choi, Chae-Young Kim, Hye Kyung Chang, Young Joo Lee, Sung-Hoon Chung

**Affiliations:** 1 Department of Pediatrics, Kyung Hee University School of Medicine, Seoul, Republic of Korea; 2 Department of Surgery, Kyung Hee University School of Medicine, Seoul, Republic of Korea; 3 Department of Obstetrics and Gynecology, Kyung Hee University School of Medicine, Seoul, Republic of Korea

**Keywords:** Respiratory distress syndrome, newborn, Pulmonary surfactant, Pulmonary surfactant associated proteins, Forkhead box M1 protein, human

## Abstract

**Introduction:** Antenatal steroid improves respiratory distress syndrome in preterm infants. The molecular mechanism of the process is not well established. The aim of this study is to investigate the possible association between antenatal steroid and fetal Forkhead box M1(Foxm1) expression.

**Materials and methods:** An animal study using mated pregnant New Zealand white rabbits and their fetuses was designed. Fourteen mother rabbits were assigned to four groups to undergo a cesarean section. In groups 1, 2, and 3, preterm pups were harvested on day 27 of gestation. In group 4, term pups were harvested on day 31. Antenatal maternal intramuscular injection was performed in groups 2 (normal saline) and 3 (betamethasone). Using qRT-PCR and Western blot, mRNA transcription and protein expression of surfactant protein (SP) A, B, C, and Foxm1 were compared between the pups of those four groups.

**Results:** Sixty two fetal rabbits were harvested. One-way ANOVA test showed higher mRNA transcription of SPs in groups 3 and 4 than groups 1 and 2. Significantly lower Foxm1 mRNA transcription and protein expression were observed in group 3 or 4 compared with group 1 or 2.

**Conclusion:** Decreased Foxm1 expression was associated in an antenatal betamethasone animal model.

## Introduction

Despite remarkable advances in neonatal intensive care, respiratory distress syndrome (RDS) is still a leading cause of death in preterm infants 1-3. In the era of improved outcomes of RDS along with introduction of pulmonary surfactant therapy and early application of non-invasive ventilation, maternal antenatal steroid has remained as another mainstay in lowering the mortality and morbidity 4. Maternal antenatal steroid confers acute changes in fetal lung tissue, which include decreased alveolar septation, mesenchymal thinning, and increased lung compliance and gas-exchange 5,6. Several mechanisms in those changes appear to be explainable, such as maternal cortisol effect, the stress response of the fetus, or the influence of chorioamnionitis 7,8.

In addition, some studies regarding embryonal development of pulmonary tissue reported the importance of the expression of Forkhead box M1 (Foxm1). They studied the association of Foxm1 with preterm rabbit lung 9. Foxm1 protein is one of extensive transcription factors sharing its homology in the Winged helix/forkhead DNA binding domain. It directly influences mitosis and its deficient hepatoblasts and cardiomyocytes showed decreased DNA replication due to accumulation of polypoid phenotype and failed to progress to mitosis in the end 10-12. Interestingly, it involves lung development. Foxm1 knock-out mice showed severe hypertrophy of arteriolar smooth muscle cells in lung tissue and deficient platelet endothelial cell adhesion molecules in peripheral pulmonary capillaries 13. In another study, conditioned deletion of Foxm1 was induced in mouse pulmonary tissues, which led to failure of lung development with decreased expression of SP-A, B, C, and D^14^. Remarkably, Foxm1 has a striking characteristic, it is enhanced during cell proliferation, and later, is extinguished when cell differentiation is over 15,16.

Therefore, in the current study, we hypothesized that there should be certain relationship between antenatal steroid and FOXM1 expression in a fetal rabbit model animal experiment.

## Materials and methods

The animal experiment was approved by the Institutional Animal Care and Use Committee of Kyung Hee University Hospital at Gangdong. All surgical procedures were performed under anesthetic agent Zoletil^®^ (tiletamine + zolazepam, 15 mg/kd). An antenatal maternal steroid injection model in preterm delivery groups was designed. Sixty two rabbit pups were harvested from 14 pregnant female New Zealand white rabbits.

### Antenatal preparation of pregnant rabbits

**Figure 1:**
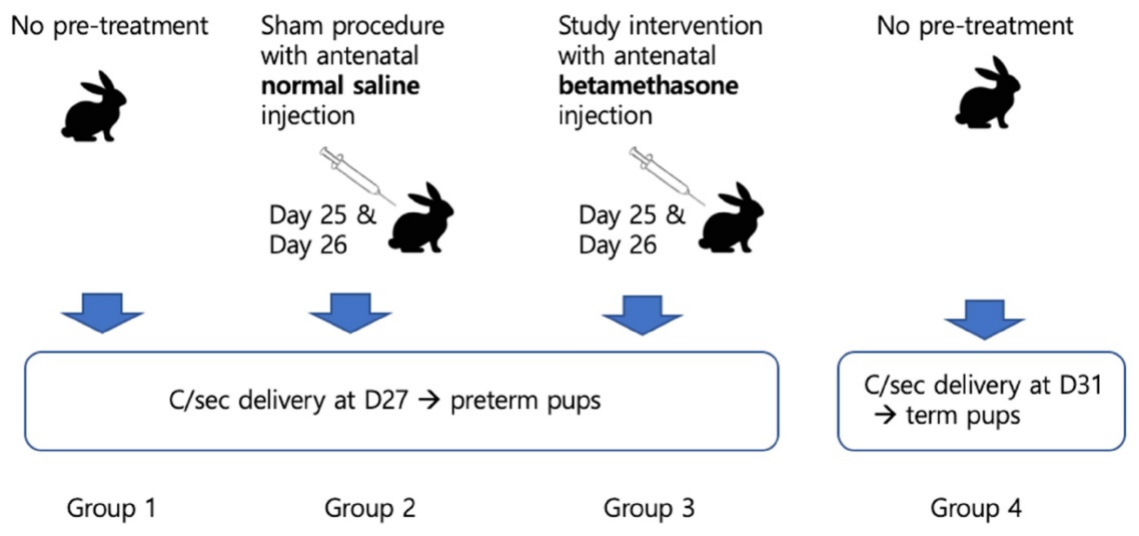
Scheme of the current study.

In terms of lung maturation, neonatal pups were categorized into four groups, basically into preterm (Group 1, 2, and 3) or term (Group 4). Delivery was scheduled by cesarean section for controlled condition on day 27 in preterm groups and day 31 in the term group. Controls were Group 1 as a preterm control and Group 4 as a term control. Mothers of Group 2 pups underwent antenatal normal saline injection on days 25 and 26, which was for the sham procedure. Mothers of Group 3 pups (study group) received an interventional antenatal betamethasone injection on days 25 and 26 (Figure 1).

The antenatal injection preparations for the maternal rabbits were as follows: Betamethasone, 0.05 mg/kg and normal saline, 0.0125 ml/kg, which were the same by volumes. The injections were only administered intramuscularly in groups 2 and 3 and the schedule for days 25 and 26 were mimicked like human protocol.

### Preparation of lung tissues from newborn rabbit pups

All the newborn pups underwent tracheostomy using an 18-gauge angiocatheter. Forced aeration of 0.5 ml was applied 10 times using a 1.0 ml syringe before obtaining the lung tissues. The lung tissues were used for the mRNA analysis, Western blot, and histologic findings. Total mRNA extractions were performed using the RNeasy® mini kit Qiagen.

### Measurement of surfactant protein (SP) A, B, C and Foxm1 level

Quantitative real-time RT-PCR (qRT-PCR) was used for measurement of the mRNA level and Western blot was used for the protein expression. SP-A, B, and C mRNA quantitation was performed according to the SYBR® Green I protocol. Primers were purchased from BIONEER (Daejeon, South Korea). The PCR protocol was performed at 95ºC for 15 seconds denaturation and 56.6ºC for 20 seconds cooling and 72ºC for 30 seconds synthesis and repeated 35 times. For Western blot, we used human Foxm1 protein antibody (Abcam®, Cambridge, UK) for primary antibody and peroxidase liked mouse antibody (Santa Cruz Biotechnology, Inc., USA) for the secondary. The concentration was based on group 4 as 1.0 and quantified by β-actin values.

### Design of rabbit Foxm1 primer

There is no known Foxm1 sequence in rabbit, therefore we designed the primer. The Foxm1 sequence of mouse (Genbank, accession number: NM_008021.4) and human (Genbank accession number: NW_003159267.1) were matched with the whole base pair of the 8^th^ chromosome of rabbit (Genbank accession number: NW_003159267.1) and then base pairs of homology were found. Four pairs of primers were designed via Primer3 website (http://frodo.wi.mit.edu/primer3/) and the most prominent band that was matched and designed with human Foxm1 sequence was selected. Finally we used anti-sense 5'-agg aaa gct gac ttg gaa ac-3' and sense 5'-gtg cat ggt ttc ttc ttc cag-3' pair.

### Histologic preparation

Some of the lung tissues of harvested pups were observed by histological method. The tissues were fixed with 10% buffered formalin and a paraffin block was made for staining Hematoxylin and Eosin (H&E). The aerated area ratio was viewed using the IBAS 2000 system (Karl Zeiss, Jena, Germany), which was calculated by ImageJ software (LOCI, University of Wisconsin). We tried to obtain the tissue slides in the midportion of the extracted specimens.

### Electron microscopic examination

Electron microscopy was also performed (FE-SEM, HITACHI S-800, Japan). We chose the best quality images from some of the slides.

### Statistical analysis

One-way ANOVA was used to compare the values for four groups. Turkey HSD was used as a posthoc test. Linear regression analysis was performed between SP mRNA and Foxm1 protein expression. Measured values were expressed as mean ± standard deviation. *P* values <0.05 were considered significant.

## Results

Sixty two rabbit pups were harvested from 14 mother rabbits (Table 1). There were no serious adverse effects such as maternal death, spontaneous preterm delivery, or fetal death. The mean birth weights of groups were 37.5±4.2, 39.3±4.2, 32.3±6.5, and 49.4±6.6 grams, respectively. Mean birth weight was significantly lower in Group 3 compared with group 2 (*p*<0.001).

**Figure 2:**
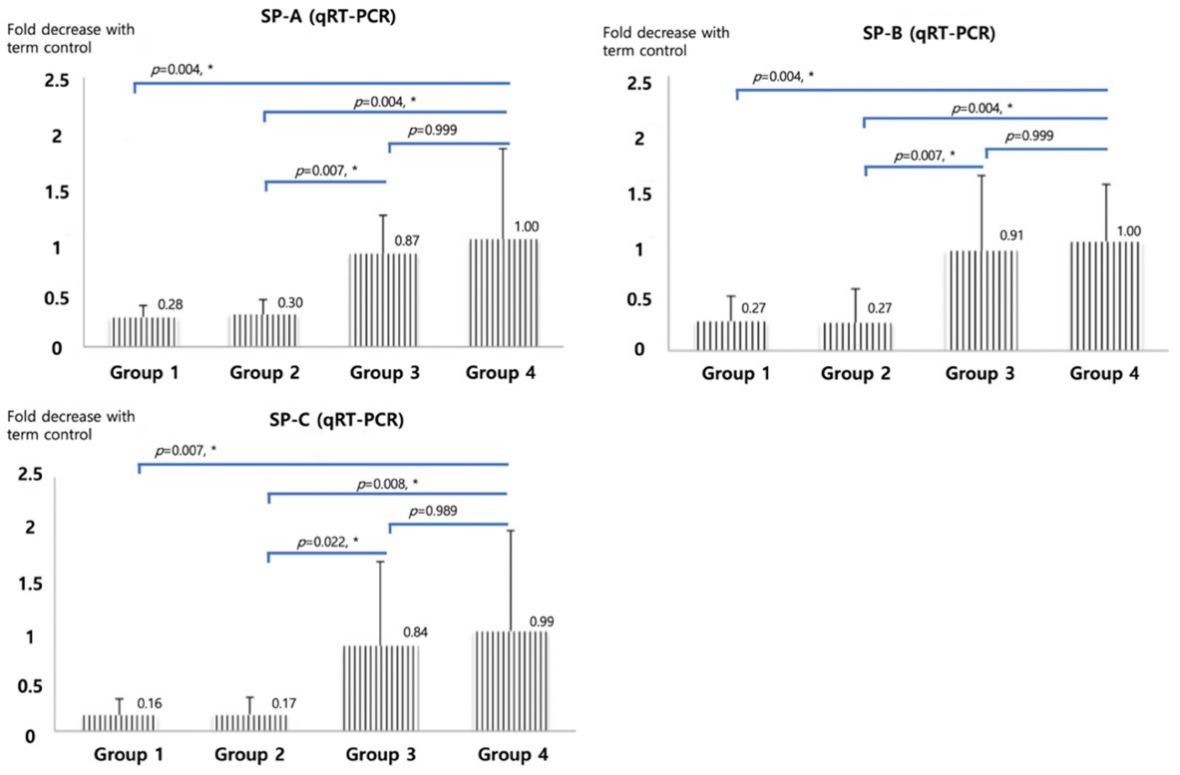
Surfactant protein A, B, and C mRNA qRT-PCR. The diagram was based on the mean values and error bar. N=12. One-way ANOVA test was used.

**Figure 3:**
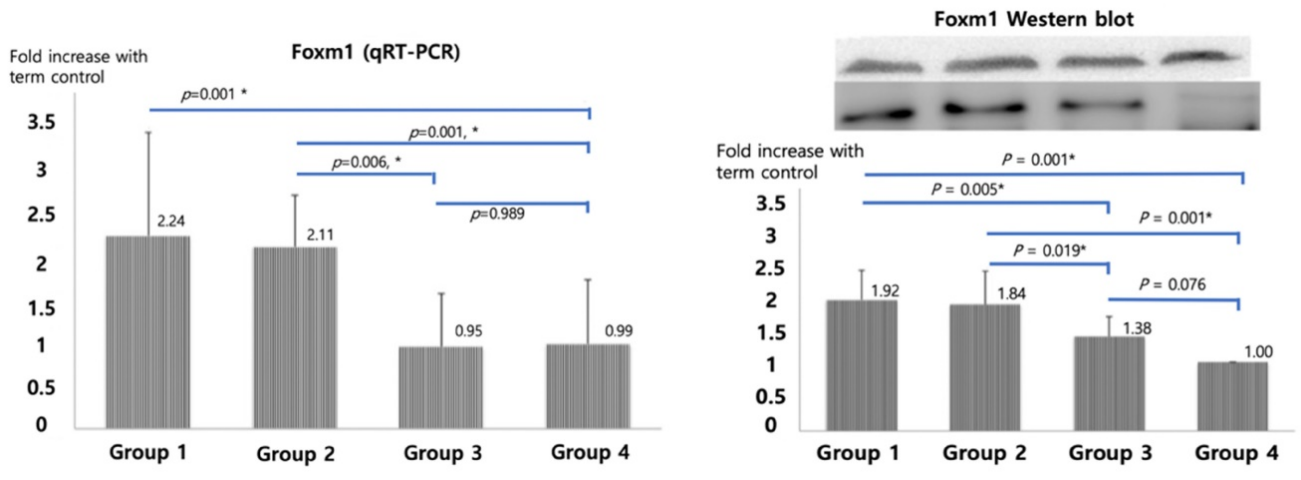
Foxm1 mRNA qRT-PCR and Western blot results. The diagram was based on the mean values and error bar. N=12. One-way ANOVA test was used.

qRT-PCR of SP-A, B and C, shown in Figure 2, were significantly higher in betamethasone (Group 3) pups compared to normal saline group (Group 2, *p*=0.007, 0.007, and 0.022) and in term (Group4) pups compared to preterm control (Group 1, *p*=0.004, 0.004, and 0.007). No significant difference was observed between groups 3 and 4. The opposite results were obtained with Foxm1 levels. Significantly higher mRNA qRT-PCT was observed in Group 1 compared to Group 4 (*p*=0.001) and in Group 2 compared to Group 3 (*p*=0.006, Figure 3). Western blot of Foxm1 in Group 1 was increased compared to Group 4 (*p*=0.001) and in Group 2 compared to Group 3 (*p=*0.019, Figure 3).

**Figure 4:**
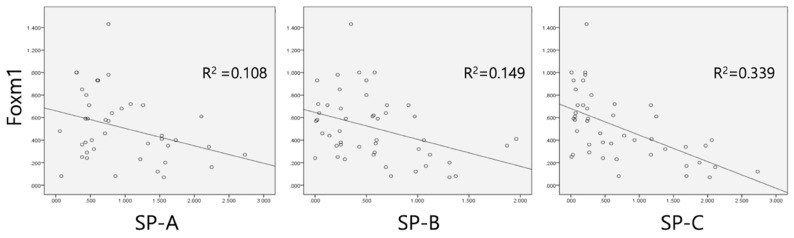
Linear regression analysis between SP-A, -B, -C and Foxm1. Negative correlation was observed between surfactant protein A, B, and C and Foxm1. Values as qRT-PCR results were used.

Linear regression performed between Foxm1 and surfactant proteins showed negative correlations between Foxm1 and SP-A (R^2^=0.108, *p*=0.036), SP-B (R^2^=0.149, *p*=0.007), and SP-C (R^2^=0.339, *p*=0.018) (Figure 4).

**Figure 5:**
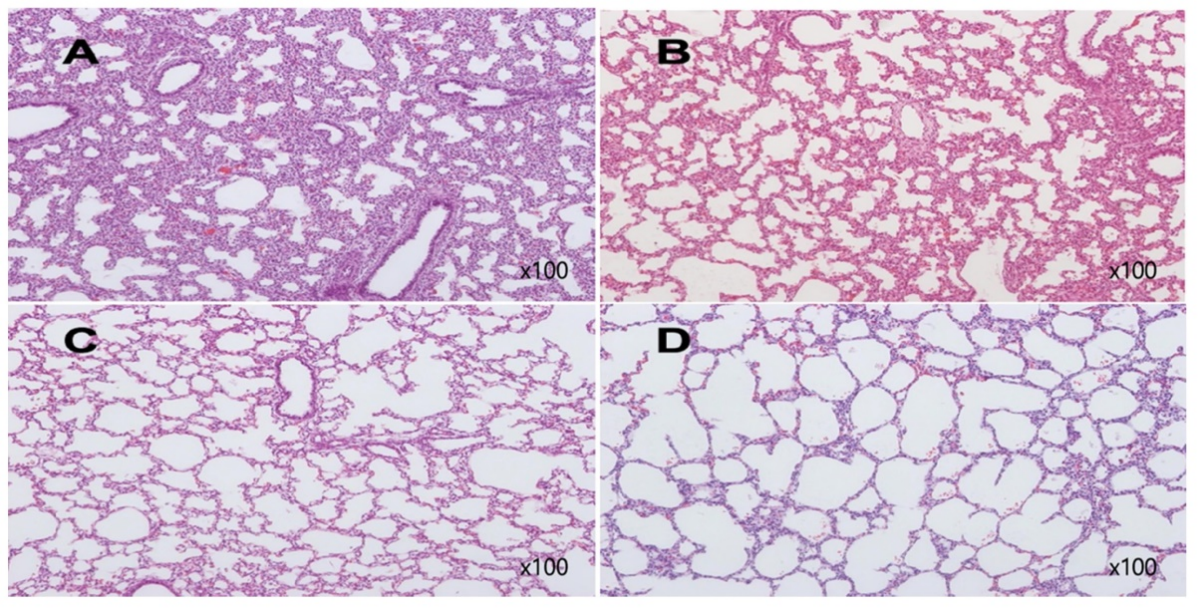
H&E stain of rabbit fetal lung tissues. A from one of group 1, Aerated area ratio (AAR)=0.27, B from one of group 2, AAR=0.3, C from one of group 3, AAR=0.85, D from one of group 4, AAR=0.95. A and B show decreased alveolarization. C shows much thinner alveolar septae comparable to those of term rabbit newborn, D.

**Figure 6:**
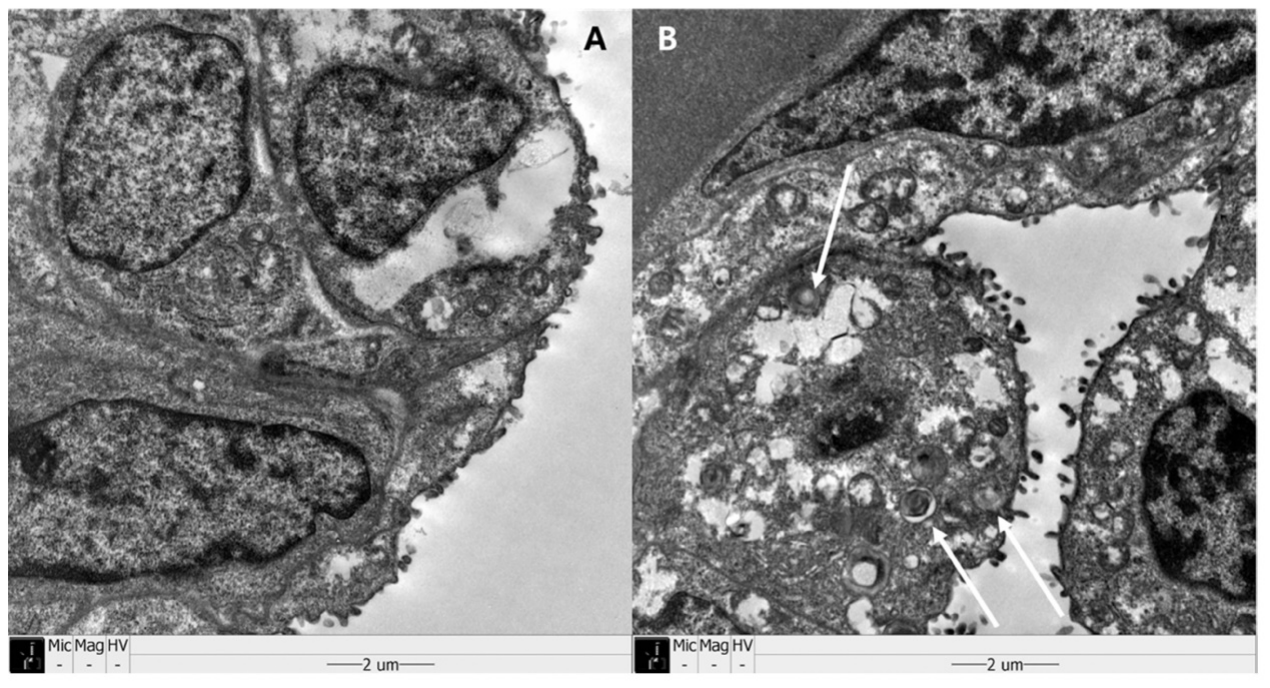
Sanning electromicroscopy (SEM) revealed type II pneumocytes. White arrows show abundant lamella bodies in one of group 3 (B), different from one of group 2 (A).

Examples of histologic findings are shown in Figures 5 and 6. Although these are just some examples of the appearance of histologic findings, we could examine the microscopy. We selected four H&E stained slides to show aerated area ratios in Figure 5. Electron microscopy showed type 2 pneumocytes from one of group 2 and group 3. Abundant lamella bodies can be seen in cytoplasm of betamethasone treated pups (Figure 6).

## Discussion

Our animal model confirmed that antenatal betamethasone induced an increase in the level of SP- A, B, and C. Numerous studies showing that antenatal steroid promotes surfactant protein synthesis have been reported 8,17. In other words, the higher the level of SPs, the larger the amount of pulmonary surfactant. This is consistent with the role of antenatal steroids in lowering severity of RDS. In terms of molecular association, we chose the Foxm1 gene. We hypothesized that Foxm1 expression has a certain relationship with the expression of SPs in the fetal lung maturation process, based on some evidence that it involves branching morphogenesis and vasculogenesis in fetal lung development 13.

It is evident that Foxm1 regulates mitosis in the process of cell cycle based on numerous studies 10-12. In terms of lung maturation, Kalin et al 14 deleted Foxm1 conditionally in respiratory epithelium and it resulted in lung tissue maturation defects. We observed that Foxm1 remained decreased in term and betamethasone group pups compared to sustained high in control preterm groups. It implicates that Foxm1 participates in the maturation process, and rapidly diminishes after the maturation is over. It can be explained by that Foxm1 promotes S-phase and M-phase entry of mitosis and facilitates cell proliferation 15,16. It is not known whether Foxm1 has a direct influence on SPs or other alveolar thinning processes. However, we could find the negative correlation between Foxm1 and SPs induced by antenatal betamethasone.

We used the preterm rabbit model, which is compatible with the RDS, artificial pulmonary surfactant, and antenatal steroid study, because it is very close to human fetal lung development 17,18. D27 corresponds to the human canalicular stage of lung development, and D31 to the saccular or alveolar stage 19. We could observe the effect of betamethasone on fetal lung by the histologic finding and induction of surfactant proteins. Interestingly, recognizable fetal weight loss was observed in the betamethasone group. In human and animal studies, antenatal steroid resulted in fetal weight loss 5,20. In our study, the average birth weight of the fetal pups of the betamethasone group was 16% lower than that of the normal saline control group (33.0±6.5 g vs. 39.3±4.2 g, *p<*0.001, Table 1). Thus, we can assume that our antenatal steroid injection to mother rabbits had pharmacological effects on their fetuses.

Our study has several limitations. The sample size is relatively small. We did not examine specific surfactant synthesis transporter regarding Foxm1. However, ours is the first study to determine the association between Foxm1 and antenatal steroid injection in an animal model.

## Figures and Tables

**Table 1:** Rabbit pups grouping.

	Group 1	Group 2	Group 3	Group 4
Pregnant rabbit	4	3	3	4
Antenatal treatment	not done	normal saline	betamethasone	not done
Delivery*	D27	D27	D27	D31
Pup	16	16	14	16
Pup weight (g)	37.5±2.49	39.30±4.20	33.00±6.54	49.44±6.56

*, delivery date after mating.
